# Reassessing AI in Medicine: Exploring the Capabilities of AI in Academic Abstract Synthesis

**DOI:** 10.2196/55920

**Published:** 2024-12-23

**Authors:** Zijian Wang, Chunyang Zhou

**Affiliations:** 1 Shandong University of Traditional Chinese Medicine College of Traditional Chinese Medicine Jinan China; 2 Department of Radiation Oncology, Qilu Hospital (Qingdao) Cheeloo College of Medicine Shandong University Qingdao China

**Keywords:** ChatGPT, AI-generated scientific content, plagiarism, AI, artificial intelligence, NLP, natural language processing, LLM, language model, text, textual, generation, generative, extract, extraction, scientific research, academic research, publication, abstract, comparative analysis, reviewer bias

In their study, Cheng and colleagues [1] delved into a comparison between academic abstracts generated by ChatGPT and those written by humans. Their aim was to evaluate the applicability of an artificial intelligence (AI) model, specifically ChatGPT 3.5, in generating abstracts for basic preclinical research. This investigation, involving 30 articles from renowned journals, revealed that the quality of ChatGPT-generated abstracts generally falls short compared to human-written counterparts, particularly in unstructured formats, occasionally leading to erroneous conclusions. The study also concluded that such AI-generated abstracts can be identified by humans. However, several aspects of this study invite further discussion.

First, the selection process for the experts involved in the study was not explicitly detailed, potentially leading to subjective biases. These biases could arise from the experts’ personal preferences, academic or cultural backgrounds, or their familiarity with the subject matter. Such biases might distort the results, causing the quality of AI-generated abstracts to be overestimated or underestimated.

Second, the study used ChatGPT 3.5 as the primary research model. However, there are concerns that ChatGPT 3.5 might not be ideally suited for processing lengthy texts, potentially leading to misunderstandings or the provision of inaccurate information [2]. Contrastingly, its accuracy might surpass that of humans when dealing with short and concise texts [3,4]. We propose that employing ChatGPT in a “layered progressive” manner for text generation could address this issue. This method involves dividing an article into smaller sections, having ChatGPT summarize each section individually, and then compiling these summaries into a cohesive whole. Such an approach is likely to yield better results than generating a summary from the entire text.

Lastly, the study concluded that blinded reviewers could accurately identify ChatGPT-generated abstracts with an accuracy of 93%. Does this conclusion actually hold practical significance? Does the realization that an abstract is AI generated influence the reviewers’ judgment of its content? In scientific research, the primary goal of writing a paper is to present research results to the readers. If AI assistance in refining an article does not alter the accuracy of the research and aids in clearer communication of research content, such practice should be deemed acceptable. A paper generated by AI and reviewed by humans, conveying high-quality research findings, deserves acceptance without bias toward its AI-generated origin.

Therefore, what needs to be avoided is the complete reliance on AI language models for labor-intensive tasks, as demonstrated in the study, where an entire article is entrusted to AI. This could lead to a lack of critical thinking and the production of low-quality or erroneous articles by human researchers. We must recognize the importance of AI language models as assistants in our writing endeavors. They can inspire our ideas and help refine our text (as researchers often lack the literary flair of writers). If the authors of the article consider the above suggestions, the conclusions of their study might change.

## Abbreviations

AI: artificial intelligence
